# Iron accumulation induced by hepcidin1 knockout accelerates the progression of aging osteoporosis

**DOI:** 10.1186/s13018-024-04535-z

**Published:** 2024-01-12

**Authors:** Lu-lin Liu, Zhong-rui Liu, Lu-jun Cao, Jun Wang, San-ming Huang, Shui-gen Hu, Yi-zhong Yang, Dong-sheng Li, Wei-wei Cao, Qing-bao Zeng, Sheng Huang, Qiong Wu, Jian-hua Xiao, Wu-yang Liu, Yao-sheng Xiao

**Affiliations:** 1 Department of Orthopedics, The First Affiliated Hospital of Gannan Medical University, No. 128, Jinling Road, Ganzhou, 341000 Jiangxi China; 2 Ganzhou Key Laboratory of Osteoporosis Research, No. 23, Qingnian Road, Ganzhou, 341000 Jiangxi China; 3 Department of Orthopedics, The People’s Hospital of Ningdu County, No. 109, Zhongshan South Road, Ningdu County, Ganzhou, 342800 Jiangxi China

**Keywords:** Iron, Hepcidin, Osteoporosis, Aged mice

## Abstract

**Objective:**

Iron accumulation is associated with osteoporosis. This study aims to explore the effect of chronic iron accumulation induced by hepcidin1 deficiency on aging osteoporosis.

**Methods:**

Iron accumulation in hepcidin1 knockout aging mice was assessed by atomic absorption spectroscopy and Perl’s staining. Bone microarchitecture was observed using Micro-CT. Hepcidin, ferritin, oxidative stress, and markers of bone turnover in serum were detected by enzyme-linked immunosorbent assay. Bone formation and resorption markers were measured by real-time quantitative PCR. Cell aging was induced by D-galactose treatment. CCK-8, flow cytometry, EdU assays, and Alizarin red staining were performed to reveal the role of hepcidin1 knockout in cell model. Iron Colorimetric Assay Kit and western blot were applied to detect iron and ferritin levels in cells, respectively.

**Results:**

In hepcidin1-knockout mice, the ferritin and iron contents in liver and tibia were significantly increased*.* Iron accumulation induced by hepcidin1 knockout caused a phenotype of low bone mass and deteriorated bone microarchitecture. Osteogenic marker was decreased and osteoclast marker was increased in mice, accompanied by increased oxidative stress level. The mRNA expression levels of osteoclast differentiation markers (RANKL, Mmp9, OPG, Trap, and CTSK) were up-regulated, while bone formation markers (OCN, ALP, Runx2, SP7, and Col-1) were down-regulated in model group, compared to wild type mice. In vitro, hepcidin1 knockdown inhibited proliferation and osteogenic differentiation, while promoted apoptosis, with increased levels of iron and ferritin.

**Conclusion:**

Iron accumulation induced by hepcidin1 deficiency aggravates the progression of aging osteoporosis via inhibiting osteogenesis and promoting osteoclast genesis.

**Supplementary Information:**

The online version contains supplementary material available at 10.1186/s13018-024-04535-z.

## Introduction

Osteoporosis is a systemic bone disease induced by low bone mass, deterioration of the microstructure of bone tissue, causing an increase of bone fragility [1]. Along with the social aging intensifies, the aging population and the incidence of osteoporosis will soar in the near future, becoming an emerging public health concern [2, 3]. It is reported that osteoporosis leads to nearly 9 million fractures annually worldwide [4]. Osteoporotic fragility fractures can induce severe pain, disability and even shortened life expectancy and hip fractures requires hospitalization, with fatalities occurring in 20% of cases, permanent impairment occurring in 50% of cases, and full recovery occurring in only 30% of cases [4]. Due to the limited efficiency and combined complications of current strategies for osteoporosis, novel treatments are urgently needed.

Primary osteoporosis includes postmenopausal osteoporosis (type I) and senile osteoporosis (type II), accounting for the major causes of osteoporosis and subsequently osteoporotic fractures in clinic [5]. Type I osteoporosis is mainly induced by the loss of estrogen, while type II osteoporosis is mainly caused by the cellular senescence associated with aging. Skeletal aging is characterized by bone loss and the accumulation of bone marrow adipose tissue, which is common in both mice and humans [6, 7]. Many factors contribute to aging of bones, such as genomic instability, telomere attrition, accumulation of reactive oxygen species (ROS), which can lead to activation of the cellular senescence program in vivo and disrupt the balance between osteogenesis and osteoclast genesis, resulting in bone loss and bone quality decline [8–10]. However, the underlying molecular mechanisms of osteoporosis caused by cellular senescence have not been studied deeply.

Senescent cells often accumulate large amounts of intracellular iron (up to 30-fold), which is accompanied by changes in the levels of iron homeostasis proteins, making ferritin a powerful biomarker of cellular senescence [11]. Iron is essential for many types of biological processes, but excess iron can damage cells and cause harmful effects on the body through iron-catalyzed oxidative stress or promoting ferroptosis, inducing cell death and various diseases [12]. Recently studies have suggested that iron may be an independent risk factor for osteoporosis [13, 14]. Osteoporosis often occurs in diseases associated with iron accumulation, such as thalassemia, hereditary hemochromatosis, sickle cell anemia, and chronic liver diseases [15–18]. Furthermore, while estrogen decreases by 90% during the menopausal transition, serum ferritin levels increase by two to three times from pre-menopause to post-menopause [19]. Notably, iron accumulation accelerates bone loss and increases the risk of fractures in healthy postmenopausal women [20]. Various in vivo models, including mice, rat, zebra fish, subjected to exogenous iron agent intervention or genetic engineering, have demonstrated that iron overload can inhibit osteoblast genesis and stimulate osteoclast genesis [21–23]. However, the effect of chronic and persistent iron accumulation on bone metabolism is still unknown, especially in elderly status.

Hepcidin, an iron reducing hormone, plays a key role in the regulation of system iron homeostasis [24]. Hepcidin interacts with ferroprotein 1, the only known iron export membrane protein, and then internalizes and degrades ferroprotein 1 to reduce the absorption of iron from the intestines to increase iron storage in organs, such as liver and spleen, thus reducing the circulating iron content [25]. Hepcidin1 (Hepc1) affects profoundly on iron metabolism, and severe iron accumulation is observed in Hepc1-knockout mice from 2 to 8 months of age [26]. Similarly, our previous study demonstrates that iron accumulation has also been observed in 7-month-old mice after Hepc1 knockout, and bone formation is significantly inhibited, causing loss of bone mass [27]. Therefore, the mechanism of the effect of iron accumulation induced by Hepc1 knockout on osteoporosis needs to be further explored.

In this study, we established Hepc1-knockout aging mice model to investigate the bone phenotype and iron status. Since oxidative stress is closely related to aging [8], and free iron can increase ROS level [28]. Therefore, we detected ROS index and antioxidant index. The expression levels of bone formation and resorption-related genes were also measured to explore the causes of bone turnover imbalance. Furthermore, cell aging model was constructed to reveal the role of Hepc1 knockout in osteogenesis in vitro. We hope to confirm the effect of chronic iron accumulation on aging osteoporosis and explore the primary mechanism to provide theoretical foundation for progression of osteoporosis.

## Materials and methods

### Animals

Wild type (WT) and Hepc1-knockout (*Hepc1*^−/−^) mice were obtained from the Cambridge-Soochow University Genome Resource Centre. We interbred Hepc1 heterozygous mice to produce Hepc1-knockout and WT offspring, obtaining approximately 25% *Hepc1*^−/−^ and WT mice, respectively, as previously described [27]. To exclude the interference of estrogen, male C57Bl/6 J mice were selected and maintained in a specific pathogen free laboratory in Soochow University, where the appropriate temperature and humidity was controlled (constant temperature of 20 ± 2 °C, humidity of 55 ± 5%). The mice were kept in 12 h-light/12 h-dark cycles with free access to standard diet and weakly acidic tap water, and trace elements in the diet with no significant difference between cohorts. All mice were killed by cervical dislocation at the age of 18 months. Blood was collected and centrifuged to obtain serum. The liver, femur, and tibia were dissected and used for relative assays. All animal experiments were performed in accordance with the Guide for the Care and Use of Laboratory Animals of the National Institutes of Health (Ethical agreement number: ECSU-201800093).

### Iron concentration in liver and bone

Iron contents in liver and tibia were analyzed by atomic absorption spectroscopy. In brief, samples of tibia were rinsed by saline to remove the bone marrow and then dried in an oven at 110 °C overnight. Dry weight was accurately measured. Dried samples were then placed at 550 °C in a muffle furnace. The obtained ash and liver samples were dissolved in aqua regia acid. The iron concentration in sample was determined by atomic absorption according to a previously published method [27].

### Perls staining of liver

Perls staining was carried out on liver tissues for observing ferric iron deposits. Liver tissues were fixed in 10% buffered formalin and embedded in paraffin. After gradient dehydration, paraffin embedding was made into paraffin sections. The paraffin slices were soaked with xylene and washed with double distilled water at 37 °C. Prussian blue staining was performed with appropriate Prussian blue staining solution and sections were incubated at 37 °C for 30 min. The slices were continue incubated at room temperature and sheltered from light for 24 h. After double distilled water immersion, the liver iron accumulation was observed under optical microscope and the representative image were collected.

### Micro-CT analysis

The left femurs were placed with gauze and scanned with a SkyScan 1172 high-resolution micro-CT scanner (SkyScan, Aartselaar, Belgium) using a 9 μm resolution, 50 kV, 500 μA, and 0.5° rotation step. Trabecular regions of interest were defined from a point of approximately 540 μm proximal to the end of the distal growth plate over 1.35 mm toward the diaphysis. Cortical regions of interest were outlined at femoral middle-diaphysis from a point of approximately 4.59 mm proximal to the end of the distal growth plate over 900 μm toward the diaphysis. Three-dimensional reconstruction and data processing were performed using the cone-beam reconstruction software CT Analyser. The global threshold was set as the lowest bone density. Calculation methods of bone parameters were performed as previously described [22].

### Measurement of hepcidin, ferritin, oxidative stress, and markers of bone turnover in serum

Serum samples were collected to analyze hepcidin (Elabscience, Wuhan, China), ferritin (Abcam, Cambridge, UK), osteogenic marker: osteocalcin (Cloud-Clone corp, Houston, US), and osteoclast marker: C-terminal telopeptide of type 1 collagen (CTX; Cloud-Clone corp) levels by enzyme-linked immunosorbent assays. Serum oxidative stress markers, malondialdehyde (MDA) and superoxide dismutase (SOD) were detected by using the MDA assay kit (Jiancheng, Nanjing, China) and SOD activity assay kit (Jiancheng), respectively. The optical density (OD) was measured at wavelengths of 532 nm and 450 nm, respectively. All the procedures were performed according to the manufacturers' instructions.

### Expression levels of bone formation and resorption-related genes

To explore the causes of bone turnover imbalance, we performed real-time quantitative PCR (RT-qPCR) to detect the expression levels of genes related to bone formation and bone resorption. Bone samples were mashed into powder using liquid nitrogen and total RNA was obtained using TRIZOL regent. RNA was reversely transcribed into cDNA using a reverse transcription kit (AM1710; Invitrogen, Carlsbad, CA, USA). cDNA (2 μg) was used for real-time PCR using ABI7500 quantitative PCR instrument (Applied Biosystems, Foster City, CA, USA) and SYBR Premix Ex Taq (#RR420A; Takara, Otsu, Shiga Japan). The obtained Ct values were analyzed using the 2^−ΔΔCt^ method, and the formula was as follows: ΔΔCt = [Ct (target gene)-CT (internal reference gene)] experimental group-[Ct (target gene)-CT (internal reference gene)] control group. The levels of mRNA expression were normalized by the expression level of *β*-actin. The primers used were listed in Table 1.

**Table 1:** Primers used for quantitative RT-PCR

Gene	Primers (Forward/Reverse)
β-actin	5′-AGATGTGGATCAGCAAGCAG-3′ 5′-GCGCAAGTTAGGTTTTGTCA-3′
OCN	5′-GGACCATCTTTCTGCTCACTCTG-3′ 5′-GTTCACTACCTTATTGCCCTCCTG-3′
ALP	5′-CCAACTCTTTTGTGCCAGAGA-3′ 5′-GGCTACATTGGTGTTGAGCTTTT-3′
Runx2	5′-AACTTCCTGTGCTCCGTGCTG-3′ 5′-TCGTTGAACCTGGCTACTTGG-3′
SP7	5′-AGGAGGCACAAAGAAGCCATAC-3′ 5′-GATGCCTGCCTTGTACCACGAGC-3′
Col-1	5′-ACGTCCTGGTGAAGTTGGTC-3′ 5′-CAGGGAAGCCTCTTTCTCCT-3′
RANKL	5′-CACCATCAGCTGAAGATAGT-3′
	5′-CCAAGATCTCTAACATGACG-3′
OPG	5′-AGTCCGTGAAGCAGGAGTG-3′
	5′-CCATCTGGACATTTTTTGCAAA-3′
Trap	5′-TACCTGTGTGGACATGACC-3′
	5′-CAGATCCATAGTGAAACCGC-3′
Mmp9	5′-TCCAGTACCAAGACAAAG-3′
	5′-TTGCACTGCACGGTTGAA-3′
CTSK	5′-GCCGTGGCGTTATACATACA-3′
	5′-CTTCCAATACGTGCAGCAGA-3′

### Cell culture

To further confirmed the effect of Hepc1 knockout on osteogenesis, we conducted cell experiments in vitro. Mouse primary bone marrow mesenchymal stem cells (mBMSCs; iCell Bioscience Inc, Shanghai, China) were cultured using primary mBMSCs basal medium supplemented with 10% fetal bovine serum and 1% penicillin/streptomycin and placed in a 37 °C -incubator containing 5% CO_2_.

### mBMSCs aging model construction and cell transfection

D-galactose (D-gal) was used to accelerate cell aging. D-gal can increase oxidizer production, accumulation of oxidative damage, changes in antioxidant enzyme activity, leading to inflammation, tissue damage, cognitive impairment, increased ROS production, neurotoxicity, and reproductive aging [29]. Age group (D-gal): mBMSCs in complete medium containing D-gal were cultured for 48 h to induce cell aging. Control group: mBMSCs were cultured in complete medium without D-gal. To investigate the effect of Hepc1, a plasmid targeting Hepc1 siRNA (si-Hepc1-1, 2, 3) and a non-targeted siRNA (siNC) as a negative control were constructed by GeneChem Biotechnology Co. Ltd (Shanghai, China) and transfected into the mBMSCs aging model to knock down the expression of Hepc1. mBMSCs were inoculated in 24-well plates (1 × 10^5^ cells/well) and cultured until 85% confluent. The cells were then transfected using the Lipofectamine™ LTX reagent (A12621; Thermofisher, Waltham, MA, USA), according to the manufacturer’s instructions.

### Cell counting Kit-8 (CCK)-8 assay

The cytotoxicity of D-gal was detected by CCK-8 kit. mBMSCs were inoculated into 96-well plates at a density of 1 × 10^4^ cells/well and treated with D-gal (0–100 g/L) for 24 h. For the detection of cell viability, mBMSCs cells of each treatment group were inoculated in 96-well plates. The CCK-8 kits (Beyotime, Shanghai, China) were used to detect cell viability following the instructions. The OD at 450 nm was measured with an enzyme label. The specific methods were as follows: (1) The 96-well plate was prepared with 100 μL of cell suspension (1 × 10^4^ cells/well) and pre-cultured in an incubator (37 °C, 5% CO_2_) for 24 h until 80% cells fused. (2) Drug treatments were carried out in different groups, with 3 compound pores in each group; (3) 96-well culture plate was incubated in the incubator for 24 h; (4) After sucking the culture medium and washing the cells twice with Dulbecco’s Phosphate-Buffered Saline, 90 μL of maintenance medium and 10 μL of CCK-8 solution were added to each well. (5) The culture plate was placed in the incubator for 2 h. (6) The OD at 450 nm was measured with an enzyme marker.

### Transfection efficiency detection

Total RNA of cells was extracted by TRIZOL regent (ThermoFisher). RNA sample (5 μL) was diluted 20 times with RNA-free enzyme ultra-pure water. The RNA concentration and the absorption values at 260 nm and 280 nm were measured by ultraviolet spectrophotometer. The samples with the OD 260/OD 280 ratio between 1.9 and 2.0 were considered as the high-purity samples, which were selected for subsequent experiments. The cDNA was synthesized using the Hiscript II QRT Supermix for qPCR reverse transcription kit (VazymE, Piscataway, NJ, USA). The cDNA template was synthesized by reverse transcription in the PCR amplification apparatus, and the reaction procedures were as follows: 25 °C for 5 min; 42 °C for 30 min; 85 °C for 5 s. The RT-qPCR experiment was performed using the ABI7500 quantitative PCR instrument (Applied Biosystems) with the following reaction conditions: pre-denatured at 95 °C for 30 s, denatured at 95 °C for 10 s, annealed at 60 °C for 30 s, and 40 cycles. GAPDH was selected as the internal reference. The obtained Ct values were analyzed by 2^−ΔΔCt^ method. The sequence of primers was shown in Additional file 1: Table S1.

### Flow cytometry

mBMSCs cells were inoculated in 6-well plates. The cells were washed with phosphate buffered saline (PBS) and collected after centrifugation. Apoptosis was detected by FITC-labeled Annexin V and propyl iodide, according to the instructions of apoptosis detection kit (Share Bio, Shanghai, China). For Annexin V/PI staining, cells were collected and resuspended with Annexin V labeled FITC (5 µL) and incubated at room temperature for 5 min. Then 10 µL proprium iodide solution and PBS (400 µL) was added and completely mixed. Apoptosis was detected and analyzed by flow cytometry.

### Osteogenic differentiation induction

mBMSCs cells were inoculated into 6-well plates. Osteogenic differentiation was induced by high-glucose minimum essential medium containing 10% fetal bovine serum, 1% L-glutamine, 1% Penicillin/Streptomycin solution, 0.25 mM ascorbic acid, 10 mM glycerol β-phosphate, and 10 nM dexamethasone. The osteogenic induction medium was replaced every 3 days and cultured at 37 °C in 5% CO_2_ for 14 d, and the morphological changes in cells were observed. According to the precipitation of calcium salts and the formation of calcium nodules, the induction of osteogenic differentiation was terminated and the staining identification was performed.

### Alizarin red staining and calcium nodule quantification

Osteoblast calcium nodules were determined by Alizarin Red S staining. mBMSCs were inoculated in a 6-well plate. After 14 d of osteogenic induction, the cells were washed with PBS, fixed with 95% ethanol for 10 min, and then washed with PBS 3 times again. Adding 2 mL 0.2% Alizarin Red S staining solution (Solarbio, Beijing, China) to each well, cells were incubated in oven at 37 °C for 30 min. After washing with distilled water and drying, cells were observed under microscope to collect images. To quantify calcium nodules, the dye was eluted with cetyl pyridinium chloride (Sigma-Aldrich, St. Louis, MO, USA) in 10 mM sodium phosphate for 30 min. Then OD value was measured at 540 nm using a microplate reader (Thermofisher).

### 5-ethynyl-2'-deoxyuridine (EdU) assay

Cell proliferation was determined using BeyoClick™ EdU-488 Assay kit (Beyotime). mBMSCs were seeded into 6-well plates and incubated overnight. EdU working solution (10 µM) was added to the medium and incubated with cells for 3 h at 37 °C. Subsequently, 4% paraformaldehyde was used to fix cells for 15 min and washed off using PBS containing 3% bovine serum albumin (Solarbio). Furthermore, the mBMSCs were permeabilized with enhanced immunostaining permeabilization buffer (Beyotime) for 15 min. Then, the EdU detection was performed according to the manufacturer’s instructions. The nuclei were stained with Hoechst 33,342 for 10 min in the dark at room temperature. Fluorescence images were obtained by Olympus microscope (Olympus, Tokyo, Japan).

### Measurement of iron content in cells

Intracellular iron content was detected using Iron Colorimetric Assay Kit (APPLYGEN, Beijing, China). Briefly, Mixture A (200 μL, prepared following the instructions) was added to cells. Cells were incubated in 60 °C water bath for 1 h. Then, cells were cultured with iron ion detection reagent (60 μL) and were incubated at room temperature for 30 min. The OD was then measured at 570 nm using a microplate reader.

### Western blot

Protein was extracted from mBMSCs using RIPA lysis buffer (Biosharp, Beijing, China) and determined using Pierce BCA Protein Assay kit (Thermo Fisher Scientific), according to the instructions. The proteins were separated using a 10% SDS-PAGE gel and subsequently transferred to the polyvinylidene fluoride membrane. After sealing polyvinylidene fluoride membrane in 5% defatted milk, primary antibody was added and incubated at 4 °C overnight. All the primary antibodies we used in this study were as follows: anti-Ferritin (1:1000; Abcam) and anti-β-actin (1:2000; Abcam). Finally, the membranes were incubated with anti-rabbit secondary antibody (1:5000; Abcam) for 1 h at room temperature after washing with PBST. The bands were scanned using Tanon 5200 Automatic chemiluminescence image analysis system (Shanghai, China).

### Statistical analysis

All the data were processed by GraphPad Prism 7.0 statistical software. The quantitative data were presented as the mean ± standard deviation. The t-test was performed to compare two groups. ANOVA was used for comparison among multiple groups, and Tukey’s multiple comparisons test was used for comparison after ANOVA. *P* < 0.05 was considered statistically significant.

## Results

### Severe iron overload in aged ***Hepc1***^*−/−*^ Mice

**Fig. 1:**
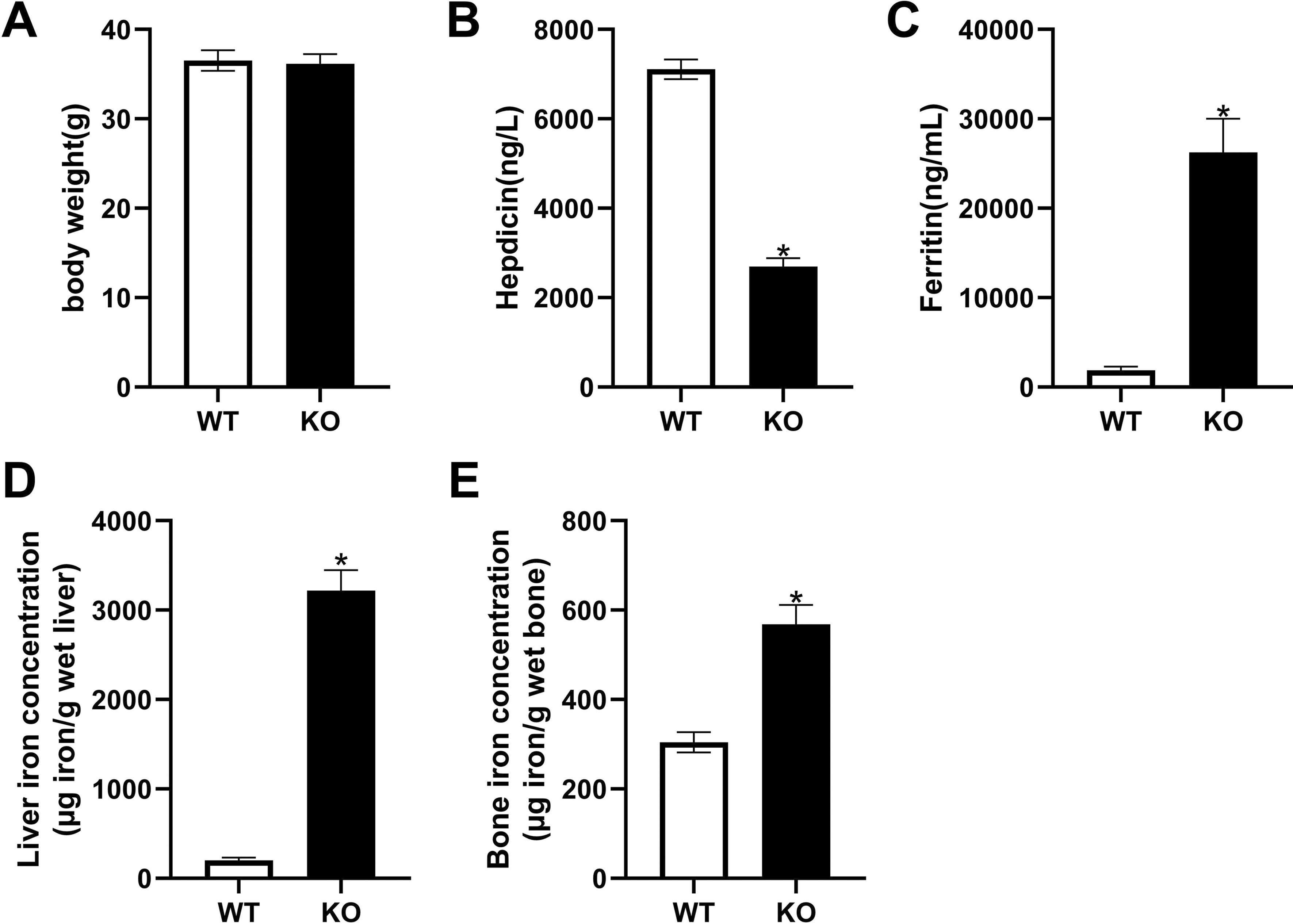
General and iron parameters in hepcidin1 knockout (KO) KO and wild type (WT) male mice at the age of 18 months. **A** body weight. **B** serum hepcidin level. **C** serum ferritin level. **D** liver iron concentration. **E** liver iron concentration. The bar graph shows the means ± standard deviation (SD). ^*^P < 0.05 *vs.* WT group

**Fig. 2:**
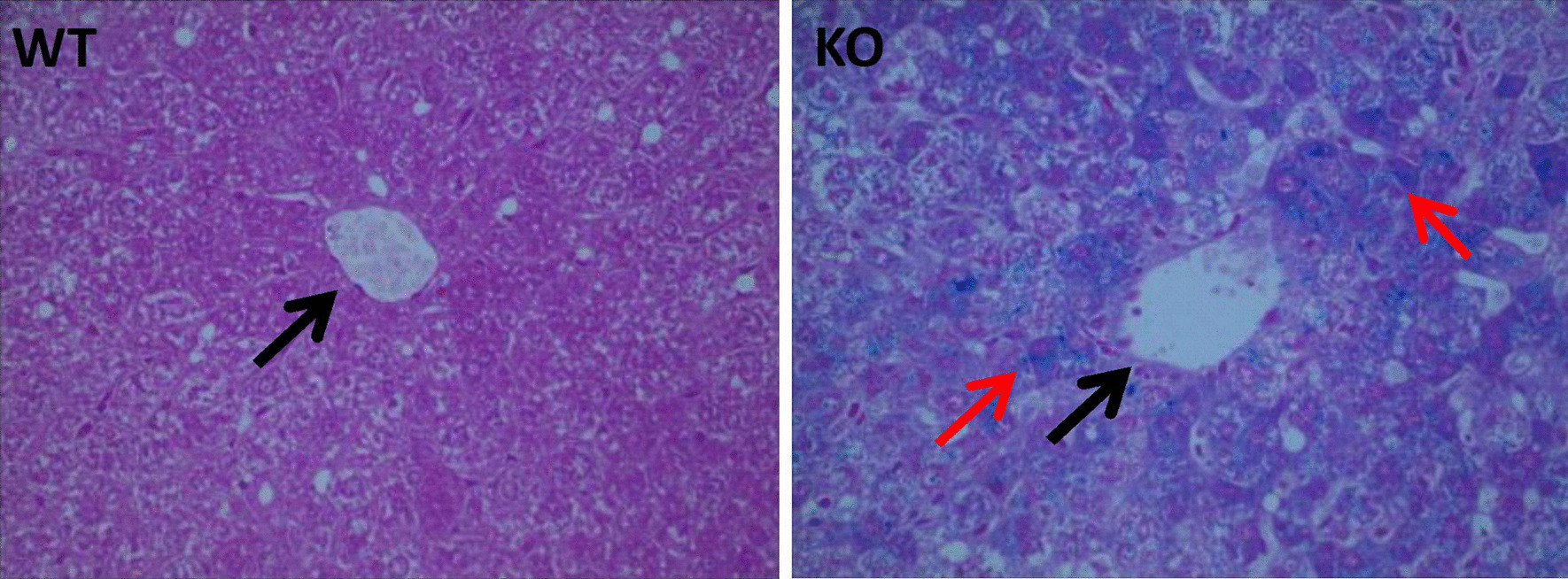
KO mice exhibit iron overload. Representative liver sections stained with Perls Prussian blue for iron shows predominantly periportal accumulation of iron of KO mice. Original magnification ×400 (black arrow is portal vein, red arrow is iron deposition)

No significant difference in appearance was observed between the WT mice and the *Hepc1-/-* mice. As shown in Fig. 1A, there were no significant differences in body weight between the *Hepc1*^−/−^ and WT mice. However, there was a significant difference between the level of serum Hepc and ferritin in *Hepc1*^−/−^ mice, compared to WT mice. Serum Hepc in *Hepc1*^−/−^ mice decreased by 62%, compared to WT mice (Fig. 1B). Serum ferritin, a classic marker of iron storage, significantly increased in in *Hepc1*^−/−^ mice, compared with WT mice (Fig. 1C). Furthermore, quantification of iron by atomic absorption showed that the iron content in the liver of *Hepc1*^−/−^ mice was about 16 times that of WT mice (Fig. 1D). The iron content in the bone was significantly higher in *Hepc1*^*-/-*^ mice, compared to WT mice (Fig. 1E). Prussian blue staining indicated that massive iron was deposited in the liver (Fig. 2).

### Abnormalities of bone microarchitecture in aged ***Hepc1***^***−/−***^*** mice***

**Fig. 3:**
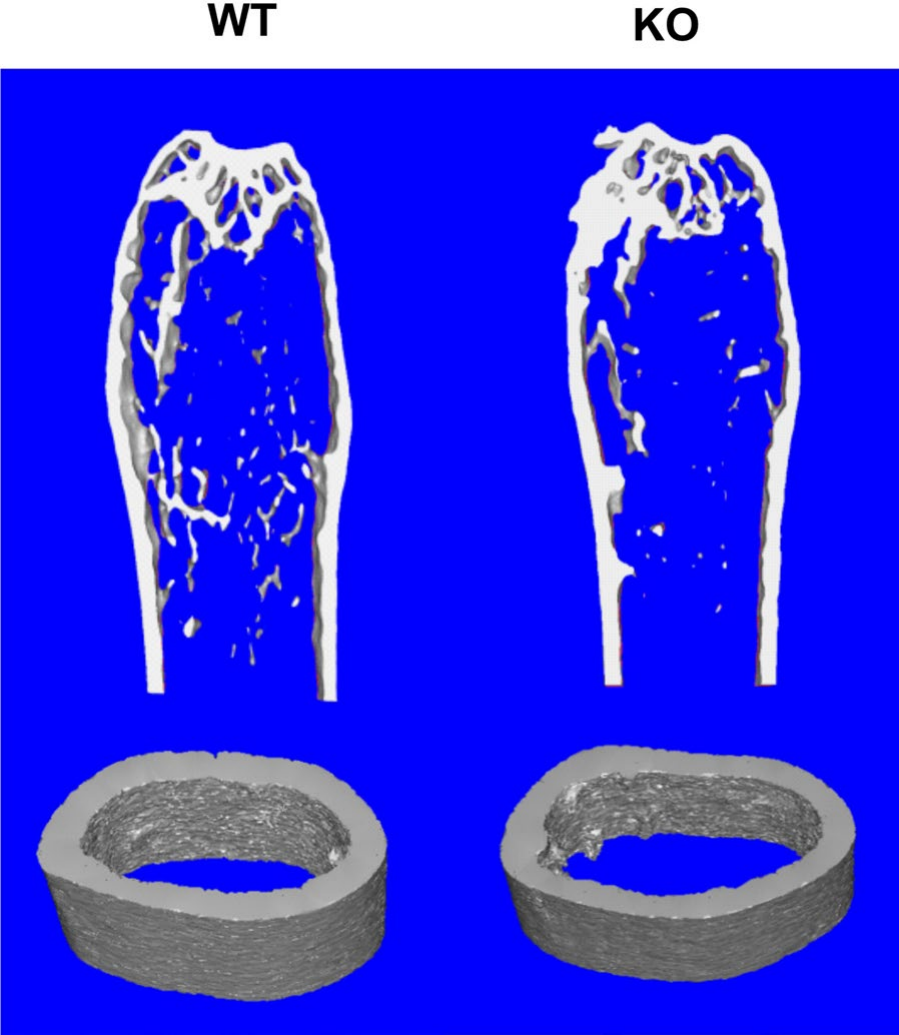
Mouse femoral metaphyseal trabecular and cortical bone architecture examined by Micro-CT

To evaluate the effect of iron overload on bone microarchitecture, micro-CT analyses of trabecular and cortical bone were performed (Fig. 3). Micro-CT analysis of the distal femur suggested that *Hepc1*^−/−^ mice had poorer trabecular bone quality than WT mice (Table 2). Bone mineral density was significantly decreased in *Hepc1*^−/−^ mice. Other measured parameters, namely, trabecular bone volume fraction, trabecular number, trabecular thickness, connectivity density, showed similar results to the bone mineral density detection results. Conversely, trabecular separation and structure model index were increased in *Hepc1*^−/−^ mice.

**Table 2:** Micro-CT analysis of mice trabecular bone (*n* = 6). Iron accumulation in KO mice markedly reduced trabecular bone mass and significantly affected other related parameters

	WT	KO
Trabecular BMD (mg/mm^2^)	0.11 ± 0.09	0.04 ± 0.016*
BV/TV (%)	9.73 ± 1.15	6.02 ± 1.63*
Trabecular number (1/mm)	0.93 ± 0.06	0.76 ± 0.10*
Trabecular thickness (µm)	89.41 ± 3.65	78.47 ± 4.67*
Tb.sp (µm)	498.25 ± 25.13	667.91 ± 22.83*
SMI	2.11 ± 0.09	2.75 ± 0.12*
ConnD (1/mm^3^)	8.59 ± 0.81	6.33 ± 0.43*
Cortical bone	–	–

The asterisks (*) indicate significant differences at *P* < 0.05

Analyses of cortical bone revealed significant difference in thickness and area of cortices between *Hepc1*^−/−^ mice and WT mice (Table 3). Similar periosteum perimeter but larger endosteal surface perimeter resulted in more confined cortical area in *Hepc1*^−/−^ mice.

**Table 3:** Micro-CT analysis of mice cortical bone (*n* = 6). Iron accumulation in KO mice significantly affected cortical bone related parameters

	WT	KO
Cortical bone thickness (mm)	0.14 ± 0.006*	0.11 ± 0.003*
Cortical area (mm^2^)	0.73 ± 0.057*	0.66 ± 0.066*
Endosteal surface perimeter	3.78 ± 0.16*	3.91 ± 0.13
Periosteum perimeter	5.25 ± 0.76	5.47 ± 0.66*

The asterisks (*) indicate significant differences at *P* < 0.05

### Unbalanced bone turnover markers and increased ROS level in aged Hepc1^***−***/***−***^ Mice

**Fig. 4:**
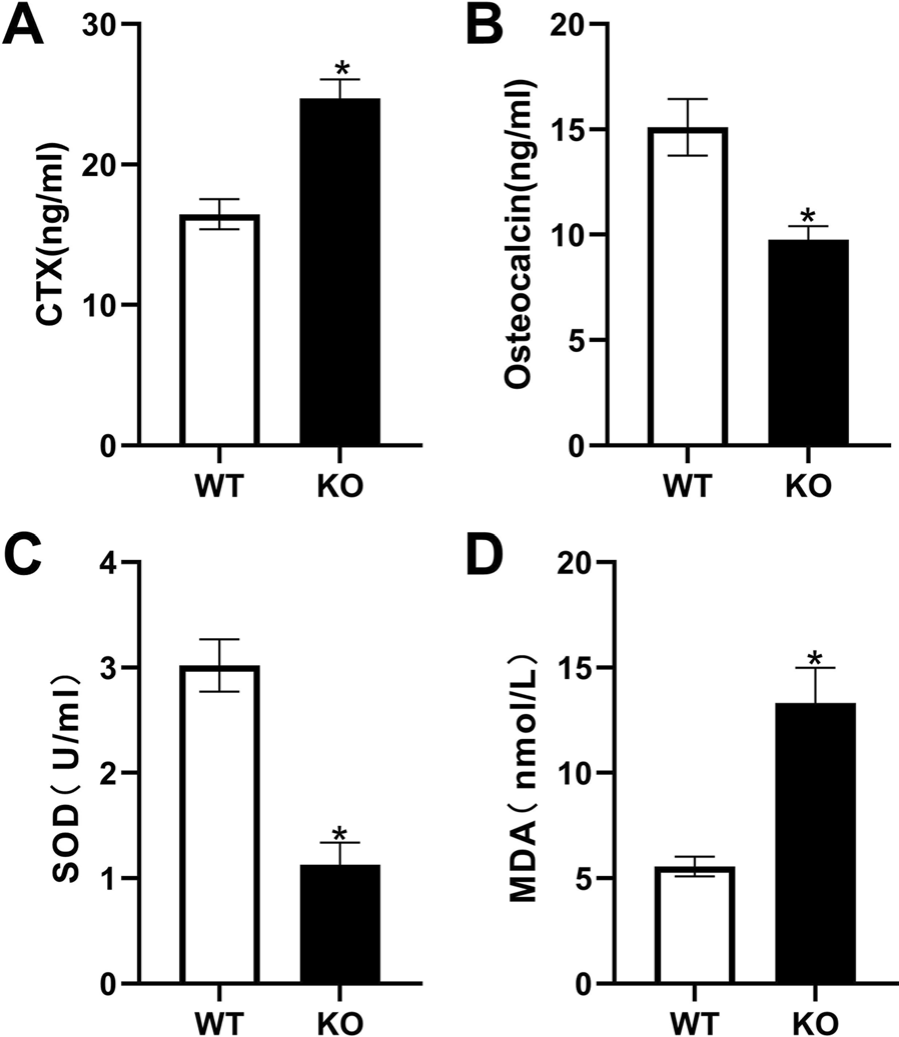
Evaluation of bone resorption, bone formation markers and oxidative stress. **A** Serum levels of the bone resorption markers C-terminal telopeptide of type 1 collagen. **B** markers of bone formation osteocalcin. **C**, **D** Levels of the oxidative stress markers superoxide dismutase and malondialdehyde. The bar graph shows the means ± SD. ^*^P < 0.05 *vs.* WT group

In *Hepc1*^−/−^ mice, the levels of CTX were significantly higher while osteocalcin concentration was significantly lower, compared to those in WT mice (Fig. 4 A and B). After measuring the levels of MDA and SOD, the results demonstrated that iron accumulation promoted the generation of MDA in *Hepc1*^−/−^ mice, while SOD showed a decreased trend, compare to WT mice (Fig. 4C and D).

**Fig. 5:**
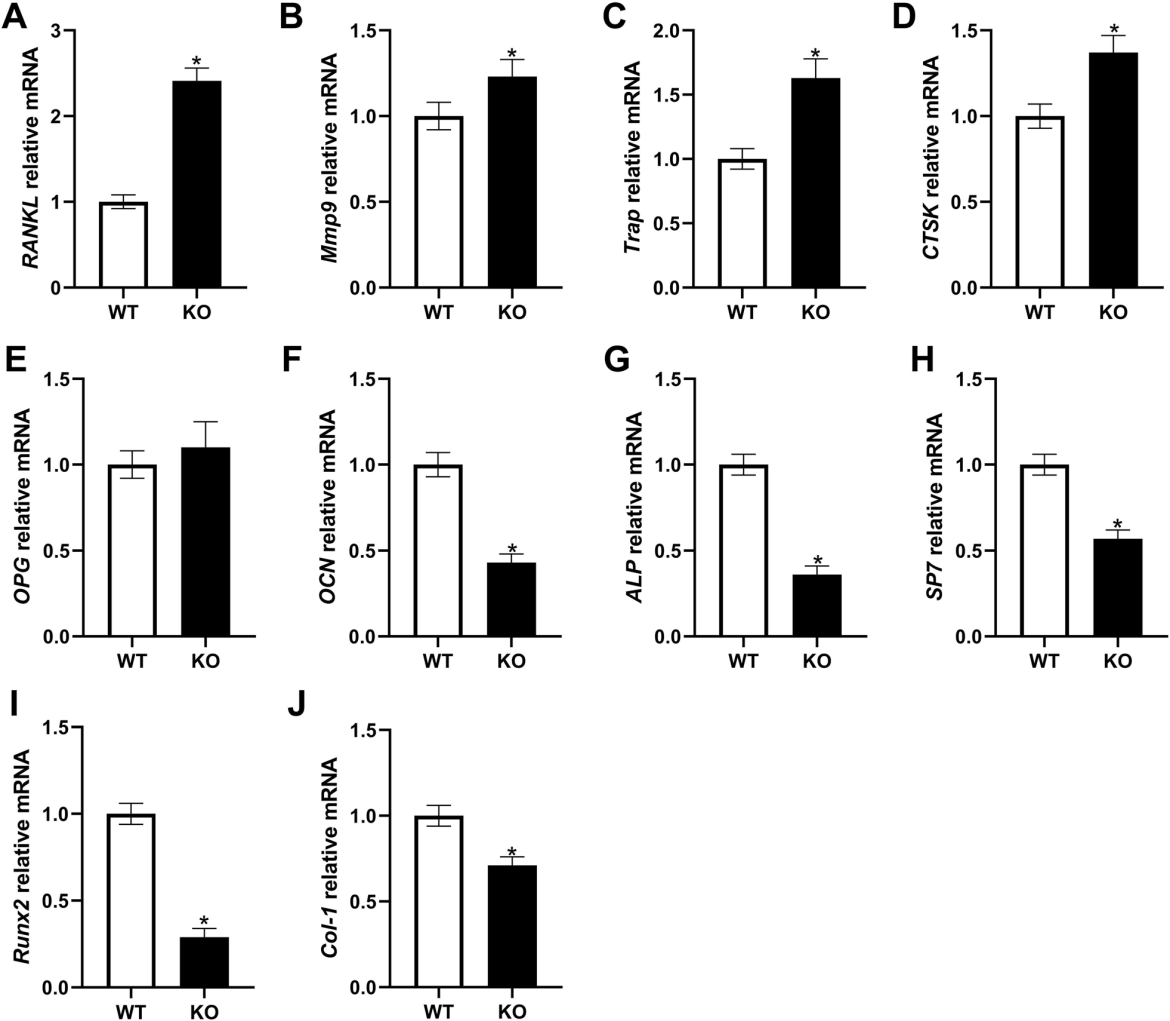
Expression of genes related to bone formation and resorption. The mRNA expression levels of **A** RANKL. **B** Mmp9. **C** Trap. **D** CTSK. **E** OPG. **F** OCN. **G** ALP. **H** SP7. **I** Runx2. **J** Col-1. The bar graph shows the means ± SD. ^*^P < 0.05 *vs.* WT group

The expression levels of osteoclast differentiation marker genes (RANKL, Mmp9, Trap, CTSK, and OPG) in the *Hepc1*^−/−^ mice were significantly up-regulated (Fig. 5A–E). Conversely, the expression levels of bone formation marker genes (OCN, ALP, SP7, Runx2, and Col-1) were down-regulated (Fig. 5F–J). These results indicated that knockout of Hepc1 significantly suppressed the differentiation ability of osteoblasts, while enhanced the function of osteoclasts.

### Hepc1 knockdown inhibits proliferation and osteogenic differentiation, while promotes apoptosis of aging mBMSCs

**Fig. 6:**
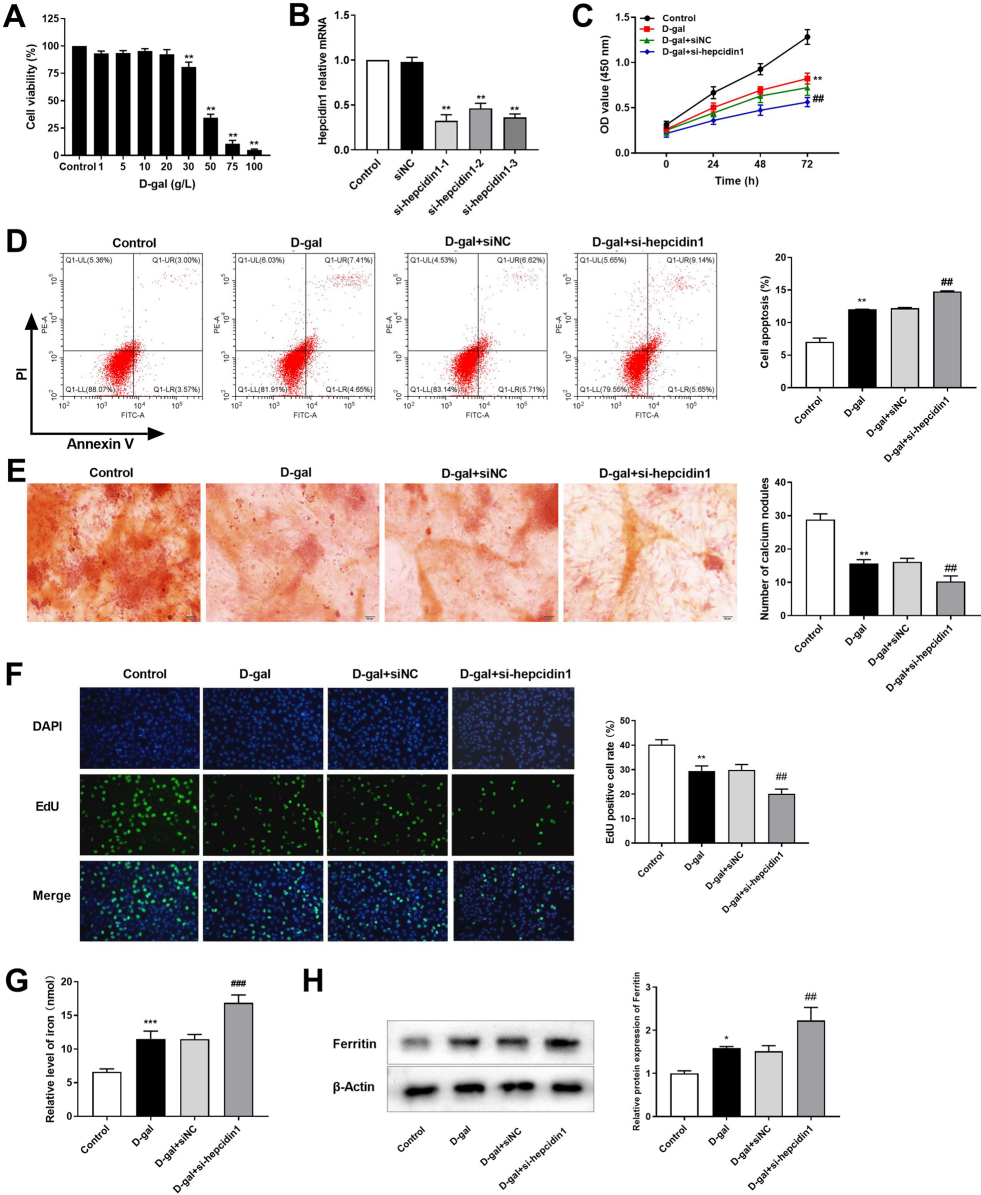
**A** The cell viability of mBMSCs treated with different concentrations of D-galactose (0–100 g/L) for 24 h was measured by CCK-8. **B** The transfection efficiency of hepcidin1 in mBMSCs was detected by real-time quantitative PCR. **C** The effect of hepcidin1 on the viability of mBMSCs determined by CCK-8. **D** Apoptosis of mBMSCs was detected by flow cytometry. **E** The changes in calcium nodules after transfection of mBMSCs were evaluated by Alizarin red staining (Amplification: 200× , Scale: 100 μm). **F** The proliferation of mBMSCs was detected by EdU assay. **G** The concentration of iron ions in mBMSCs was detected by Iron Colorimetric Assay Kit. **H** The expression level of ferritin protein was detected by western blot. ^*^P < 0.05, ^**^P < 0.01, ^***^P < 0.001 *vs.* Control group; ^#^*P* < 0.05, ^##^*P* < 0.01, ^###^*P* < 0.001 *vs.* D-gal + siNC group

After treatment with different concentrations of D-gal (0–100 g/L), the cell viability decreased with a concentration-dependent manner. Low doses of D-gal could induce the aging of mBMSCs without cytotoxicity. Moreover, after 24 h treatment with 20 g/L D-gal, there was no significant difference in cell viability, compared with the control group, and the cell viability was significantly decreased after 30 g/L D-gal concentration treatment (Fig. 6A). Therefore, the dose of 20 g/L was the optimal concentration of D-gal for model construction.

In mBMSCs, there was no significant difference in the expression level of Hepc1 between the control group and the siNC group. Compared with the siNC group, the expression levels of Hepc1 in the si-Hepc1-1, 2, 3 groups were significantly decreased (Fig. 6B).

CCK-8 results suggested that D-gal treatment suppressed cell viability, compared with the control group, while knockdown of Hepc1 resulted in a decreased cell viability in mBMSCs aging models (Fig. 6C). Flow cytometry results demonstrated the apoptosis rate in D-gal group was higher than that in control group, and knockdown of Hepc1 significantly promoted the inhibitory effect of D-gal treatment on apoptosis (Fig. 6D). Alizarin red staining revealed that D-gal treatment inhibited osteogenic differentiation, and knockdown of Hepc1 in mBMSCs significantly reduced the formation of osteogenic calcium nodules (Fig. 6E). Additionally, EdU results revealed that the EdU-positive cell rate of mBMSCs in D-gal group was significantly decreased, compared with the control group. Knockdown of Hepc1 significantly reduced the number of EdU-positive cells, compared with the D-gal + siNC group (Fig. 6F). The level of iron was apparently higher in D-gal group than that in the control group. Moreover, the level of iron in D-gal + si-Hepc1 group was evaluated, compared to the D-gal + si-NC group (Fig. 6G). Western blot results suggested that the expression level of ferritin protein was increased in D-gal group, compared to the control group, and knockdown of Hepc1 increased the expression level of ferritin in aging mBMSCs, compared to the D-gal + si-NC group (Fig. 6H).

## Discussion

With aging, the balance between bone resorption and bone formation is disrupted, leading to a decrease in bone density and destruction of bone microstructure [30, 31]. Iron accumulation is associated with cell aging, and many studies suggest that iron accumulation is closely related to osteoporosis [14, 19, 20]. In this study, we explored the effect of iron accumulation on bone metabolism. We found that bone contained excess iron and an increased hepatic iron concentration was observed *in Hepc1*^*−/−*^ mice. Bone mass parameters suggested that iron deposition in *Hepc1*^−/−^ mice was related to bone loss. The microstructure of trabecular bone was deteriorated, which led to the loss of connectivity and complexity of trabecular networks, as well as a decrease in cortical bone thickness and area. Iron accumulation induced osteopenia by enhancing bone resorption while simultaneously abrogating bone formation, as well as promoting the generation of MDA while inhibiting SOD in *Hepc1*^*−/−*^ mice. In mBMSCs aging models, Hepc1 knockdown inhibited proliferation and osteogenic differentiation, while promoted apoptosis, with increased levels of iron and ferritin.

We demonstrated that in aged mouse model, mice with overloaded iron caused by hepcidin deficiency had a deteriorated bone phenotype, which was associated with imbalance of bone turnover and increased ROS level. In this model, ROS level in the mice increased significantly, and this could be for two main reasons. On the one hand, it can be stimulated by iron-mediated Fenton reaction [28]. On the other hand, ROS also accumulates with aging: Oxidative stress occurs when the body’s oxidative system exceeds its antioxidant system, which is considered to be an important factor in aging and disease [8]. The pathogenesis of postmenopausal osteoporosis is mainly due to aging, which is caused by the accumulation of ROS [32]. The main effect of ROS on osteoblasts is inducing cell apoptosis by altering the permeability of mitochondrial membranes and releasing internal apoptotic factors [33, 34]. Conversely, ROS benefits to osteoclast genesis and differentiation by activating essential pathways involved in osteoclast function including the MAPK, PI3K, and nuclear factor kappa-B pathways [35]. Taken together, iron accumulation could cause a deteriorated bone phenotype, which might be related to oxidative stress.

We found that there were significantly lower serum levels of osteocalcin in *Hepc1-/-* mice than those in WT mice, and the serum levels of CTX-1 were also notably higher, indicating that increased osteoclast activity caused by iron accumulation may also be involved in the development of osteoporosis, which is consist with previous observations in the iron accumulation model either by extraneous iron intervention [36] or by genetic engineering [37]. Osteoclasts are originated from myeloid cells of the monocyte/macrophage lineage. Following the differentiation and formation of mononuclear pre-osteoclasts, they fuse and form the multinuclear mature osteoclasts, and mature osteoclasts resorb bone matrix mainly by releasing hydrogen ions to dissolve the inorganic bone matrix and secreting proteolytic enzymes to degrade bone matrix proteins [38]. Excess exposure of iron significantly increases the gene and protein expression levels of RANKL in MLO-Y4 osteocyte-like cells in mice, causing an increase in osteoclast formation and bone resorption capacity [39]. In Irp2 knockout mice, bone iron deficiency and reduced hepatic iron are observed and the expression levels of osteoblast-active genes, such as Balp, BGP, and Col I α1 are significantly decreased in bone tissue, while the expression of osteoclast-active genes, including Ctsk and Trap, are significantly increased [40], which is consist with our results. Studies of primary mouse progenitor cells and mouse osteoblast cell lines have shown that iron exposure impairs osteoblast genesis, differentiation, and function by regulating gene expression [41, 42]. Simultaneously, iron overload induces apoptosis of osteoblast cells via eliciting mitochondrial dysfunction [43]. Moreover, iron accumulation inhibits “bone formation and angiogenic coupling” by evaluating the levels of mTOR [22]. In conclusion, excess iron has a direct effect on osteoblast function and may reduce bone formation.

This research also has some limitations. For instance, due to the limited specimens and experimental equipment, we only used micro-CT to measure bone quality parameters and we did not perform micro-CT analysis on vertebrae. Moreover, the osteoclast genesis should be further assessed by TRAP or actin filament staining. We will supplement more detection of more indicators to evaluate bone quality, vertebrae quality, and osteoclast genesis to refine our research in the future.

In summary, our data supported a conclusion that chronic iron accumulation caused by Hepc1 deficiency accelerated the progression of osteoporosis in vivo and vitro. We found that *Hepc1*^*−/−*^ aged mice had a phenotype of lower bone mass and deteriorated bone microstructure. Iron accumulation inhibited osteogenesis and promoted osteoclast genesis, which may be related to increased ROS levels. Our research shed light on the potential mechanism of the effect of iron accumulation on bone metabolism in aging, which can provide a promising intervention for osteoporosis.

### Supplementary Information

**Additional file 1: Table S1.**

## Data Availability

All data in the manuscript are available through the responsible corresponding author.
