# Supplementary material for: Iron accumulation induced by hepcidin1 knockout accelerates the progression of aging osteoporosis
Source: J Orthop Surg Res. 2024 Jan 12;19:59. doi: 10.1186/s13018-024-04535-z (PMC10785403; doi:10.1186/s13018-024-04535-z)
Supplement: Supplementary file 1 — Additional file 1: Table S1. [file 13018_2024_4535_MOESM1_ESM.docx]

Table S1. The sequence of primers

| Name | Sense | Antisense |
| --- | --- | --- |
| siNC | UUCUCCGAACGUGUCACGUTT | ACGUGACACGUUCGGAGAATT |
| si-hepcidin1-1 | CAACAGAUGAGACAGACUACA | UAGUCUGUCUCAUCUGUUGAU |
| si-hepcidin1-2 | UAGUCUGUCUCAUCUGUUGAU | UUUAUUUCAAGGUCAUUGGUG |
| si-hepcidin1-3 | GAAGGAAGAGAGACACCAACU | UUGGUGUCUCUCUUCCUUCUC |
| GAPDH | CTCATGACCACAGTCCATGC | TTCAGCTCTGGGATGACCTT |
| Hepcidin1 | TTCCCAGTGTGGTATCTGTTGC | GGTCAGGATGTGGCTCTAGGC |
